# Rheumatoid arthritis study of the Egyptian College of Rheumatology (ECR): nationwide presentation and worldwide stance

**DOI:** 10.1007/s00296-022-05258-2

**Published:** 2023-01-08

**Authors:** Tamer A. Gheita, Hala A. Raafat, Samah A. El-Bakry, Ahmed Elsaman, Hanan M. El-Saadany, Nevin Hammam, Iman I. El-Gazzar, Nermeen Samy, Nora Y. Elsaid, Suzan S. Al-Adle, Samar Tharwat, Amira M. Ibrahim, Samar M. Fawzy, Nahla N. Eesa, Rawhya El Shereef, Faten Ismail, Mervat I Abd Elazeem, Enas A. Abdelaleem, Amany El-Bahnasawy, Zahraa I. Selim, Nada M. Gamal, Maha Nassr, Samah I. Nasef, Abdel Hafeez Moshrif, Shereen Elwan, Yousra H. Abdel-Fattah, Marwa A. Amer, Doaa Mosad, Eman F. Mohamed, Dina F. El-Essawi, Hanan Taha, Mohamed N. Salem, Rasha M. Fawzy, Maha E. Ibrahim, Asmaa Khalifa, Nouran M. Abaza, Ahmed M. Abdalla, Amany R. El-Najjar, Noha A. Azab, Hanan M. Fathi, Khaled El-Hadidi, Tahsin El-Hadidi, The Egyptian College of Rheumatology (ECR) Rheumatoid Arthritis Study Group

**Affiliations:** 1 Rheumatology Department, Faculty of Medicine, Cairo University, Cairo, Egypt; 2 Internal Medicine Department, Rheumatology Unit, Faculty of Medicine, Ain-Shams University, Cairo, Egypt; 3 Rheumatology Department, Faculty of Medicine, Sohag University, Sohag, Egypt; 4 Rheumatology Department, Faculty of Medicine, Tanta University, Gharbia, Egypt; 5 Rheumatology Department, Faculty of Medicine, Assuit University, Assuit, Egypt; 6 Internal Medicine Department, Rheumatology Unit, Faculty of Medicine, Mansoura University, Dakahlia, Egypt; 7 Rheumatology Department, Faculty of Medicine, Kafr El-Skeikh University, Kafr El-Shaikh, Egypt; 8 Rheumatology Department, Faculty of Medicine, Minia University, Minia, Egypt; 9 Rheumatology Department, Faculty of Medicine, Beni-Suef University, Beni-Suef, Egypt; 10 Rheumatology Department, Faculty of Medicine, Mansoura University, Dakahlia, Egypt; 11 Rheumatology Department, Faculty of Medicine, Fayoum University, Fayoum, Egypt; 12 Rheumatology Department, Faculty of Medicine, Suez-Canal University, Ismailia, Egypt; 13 Rheumatology Department, Faculty of Medicine, Al-Azhar University, Assiut, Egypt; 14 Rheumatology Department, Faculty of Medicine, Alexandria University, Alexandria, Egypt; 15 Internal Medicine Department, Rheumatology Unit, Faculty of Medicine (Girls), Al-Azhar University, Cairo, Egypt; 16 Internal Medicine Department, Rheumatology Unit (NCRRT), Atomic Energy Authority (AEA), Cairo, Egypt; 17 Internal Medicine Department, Rheumatology Unit, Faculty of Medicine, Beni-Suef University, Beni-Suef, Egypt; 18 Rheumatology Department, Faculty of Medicine, Benha University, Kalyoubia, Egypt; 19 Rheumatology Department, Faculty of Medicine, Ain Shams University, Cairo, Egypt; 20 Rheumatology Department, Faculty of Medicine, Aswan University, Aswan, Egypt; 21 Rheumatology Department, Faculty of Medicine, Zagazig University, Sharkia, Egypt; 22 Rheumatology Department, Military Academy, Agouza Rheumatology Center, Giza, Egypt

**Keywords:** Rheumatoid arthritis, Age at onset, Gender, Biologics, Egypt, Multicenter

## Abstract

To depict the spectrum of rheumatoid arthritis (RA) in Egypt in relation to other universal studies to provide broad-based characteristics to this particular population. This work included 10,364 adult RA patients from 26 specialized Egyptian rheumatology centers representing 22 major cities all over the country. The demographic and clinical features as well as therapeutic data were assessed. The mean age of the patients was 44.8 ± 11.7 years, disease duration 6.4 ± 6 years, and age at onset 38.4 ± 11.6 years; 209 (2%) were juvenile-onset. They were 8750 females and 1614 males (F:M 5.4:1). 8% were diabetic and 11.5% hypertensive. Their disease activity score (DAS28) was 4.4 ± 1.4 and health assessment questionnaire (HAQ) 0.95 ± 0.64. The rheumatoid factor (RF) and anti-cyclic citrullinated peptide (anti-CCP) were positive in 73.7% and 66.7% respectively. Methotrexate was the most used treatment (78%) followed by hydroxychloroquine (73.7%) and steroids (71.3%). Biologic therapy was received by 11.6% with a significantly higher frequency by males vs females (15.7% vs 10.9%, *p* = 0.001). The least age at onset, F:M, RF and anti-CCP positivity were present in Upper Egypt (*p* < 0.0001), while the highest DAS28 was reported in Canal cities and Sinai (*p* < 0.0001). The HAQ was significantly increased in Upper Egypt with the least disability in Canal cities and Sinai (*p* = 0.001). Biologic therapy intake was higher in Lower Egypt followed by the Capital (*p* < 0.0001). The spectrum of RA phenotype in Egypt is variable across the country with an increasing shift in the F:M ratio. The age at onset was lower than in other countries.

## Introduction

Rheumatoid arthritis (RA) is a chronic systemic autoimmune disease primarily affecting small synovial joints usually symmetrically. Symptoms for more than 6 months establish the diagnosis of RA [1]. An intricate network of cytokines and cells trigger synovial cell proliferation and cause damage to both cartilage and bone [2].

Alone the laboratory test for RA cannot confirm a diagnosis that is commonly challenging. A complete clinical approach is necessary to diagnose and avoid debilitating joint damage [1]. Yet, auto-antibodies signify a hallmark of RA, with the rheumatoid factor (RF) and anti-cyclic citrullinated (anti-CCP) peptides being the most acknowledged. Seropositive patients present a certain disease course. With the recent improvements in diagnosis and the discovery of new autoantibodies, the group of seronegative patients is persistently shrinking [3]. Using applicable disease activity measures can help in clinical practice to take on treat-to-target strategies in RA patients [4]. There has been a rising importance for the early and demanding diagnosis and treatment of RA with the goal of reducing disability and mortality [5].

To improve the clinical outcome in RA, various therapeutic approaches are required [1], although current management recommendations may still support a 'one-size-fits-all' treatment strategy [6]. Early treatment with disease-modifying anti-rheumatic drugs (DMARDs) is standard, yet many patients progress to disability with substantial morbidity over time [1]. The arrival of biologics has changed the treatment of RA due to their remarkable impact on disease manifestations and their ability to diminish joint damage [5]. With the development of biologics and Janus kinase (JAK) inhibitors [2], these agents are being used by a rising number of patients including those with a mild disease. However, cost and safety issues remain key determinant [2, 5]. Personalized medicine is necessary to select special treatment strategies for certain clinical or molecular phenotypes of patients [6] and key factors of RA disease such as epidemiology, clinical presentations and treatment options should be presented.

In the milieu of the restricted information on the epidemiology and treatment patterns of RA across Egypt, the aim of the present study was to present the spectrum of RA in Egypt and compare it to other studies from around the world to provide broad-based characteristics to this particular population.

## Patients and methods

### Study population and design

This cross-sectional study included a large cohort of 10,364 adult RA patients (new and existing cases) fulfilling the American College of Rheumatology/European League Against Rheumatism (ACR/EULAR) classification criteria [7] that were recruited from 26 specialized rheumatology departments and centers representing 22 major governorates across the country by members of the Egyptian College of Rheumatology (ECR) during the period from September 2018 till December 2021. Any patients with another rheumatic disease or below the age of 18 were excluded. The patients’ in the corresponding university-teaching hospitals provided informed consents to participate and the study was approved by the local ethics committee, in accordance to the 1964 Helsinki declaration and its later amendments.

### Measures and outcomes

Patients were subjected to full history taking and clinical examination. Juvenile-onset RA (JoRA) cases were considered for those who developed the disease before the age of 18 years. It is noteworthy that co-morbidities or manifestations relied on the records of the files. Presence of rheumatoid factor (RF) and/or anti-cyclic citrullinated peptide (anti-CCP) were determined. The use of medications to treat RA was described. Disease activity score (DAS28) [8] and health assessment questionnaire (HAQ) [9] were assessed.

### Statistical analysis

Data were collected on a standardized data sheet and stored in an electronic database. Data missing completely at random (MCAR) as for the RF, anti-CCP and anti-nuclear antibody (ANA) positivity was handled by running a complete-case analysis (CCA), where all persons with missing values were excluded from the analysis of this test and imputation was not used. Statistical Package for Social Sciences (SPSS) version 25 was used. Variables were presented as frequencies and percentages or mean and standard deviation. A comparison was done using Chi-square test, Mann Whitney* U* tests or analysis of variance (ANOVA).* P* value < 0.05 was considered significant.

## Results

The study included 10,364 RA patients recruited from 22 governorates across Egypt. Their mean age was 44.8 ± 11.7 years. They were 8750 females and 1614 males (F:M 5.4:1). Characteristics of the patients and gender differences are presented in Table 1. 209 (2%) were Jo-RA. Steroids were received by 71.3% of the patients. DMARDs were received in the following descending frequency: methotrexate (MTX) (78%), hydroxychloroquine (HCQ) (73.6%), leflunomide (LFN) (54.8%), sulfasalazine (SAZ)(37.2%), cyclophosphamide (CYC) (2.4%), azathioprine (AZA)(2%), cyclosporine A (CSA)(0.5%) and mycophenolate mofetil (MMF)(0.46%). Steroids and DMARDs received were comparable between genders except for HCQ (male: 77.6% vs females 73%; *p* = 0.002). Biologic therapy was received by 11.6% with a significantly higher frequency by males vs females (15.7% vs 10.9%, *p* = 0.001). Biologic therapies received were etanercept (30.4%), adalimumab (18.4%), golimumab (14%), rituximab (7.9%), infliximab (3.3%), tofacitinib (1.6%), certolizumab (1%), upadacitinib (0.8%), baricitinib (0.39%), abatacept (0.39%) and undefined (17.8%). Patients also received low dose aspirin (4.6%), colchicine (1.3%) and oral anticoagulants (1.1%).

**Table 1:** Characteristics of the rheumatoid arthritis patients and gender differences: demographic features, co-morbidities, manifestations, laboratory investigations, functional status and disease activity

Parameter *n* (%) or mean ± SD	Rheumatoid arthritis patients			*p*
	All (*n* = 10,364)	Females (*n* = 8750)	Males (*n* = 1614)	
Age (years)	44.8 ± 11.7	44.4 ± 11.6	47.1 ± 12.1	** < 0.0001**
Female:Male	5.4:1	–	–	–
Disease duration (years)	6.4 ± 6.03	6.4 ± 6	6.5 ± 6.4	0.66
Age at onset (years)	38.4 ± 11.6	38 ± 11.4	40.7 ± 12.3	** < 0.0001**
BMI	28.5 ± 5.3	28.5 ± 5.3	28.6 ± 5.5	0.89
Smoking	849 (8.2)	213 (2.4)	636 (39.4)	** < 0.0001**
Married	9458 (91.1)	7993 (91.3)	1465 (90.8)	0.64
Comorbidity				
Diabetes mellitus	833 (8)	689 (7.8)	144 (8.9)	0.22
Hypertension	1194 (11.5)	995 (11.4)	199 (12.3)	0.36
HCV	88 (0.85)	72 (0.82)	16 (1)	0.55
Bronchial asthma	68 (0.66)	63 (0.72)	5 (0.31)	**0.01**
Thyroid dysfunction	185 (1.8)	178 (2)	7 (0.43)	** < 0.0001**
*Family hx RA* (1st degree)	155 (1.5)	136 (1.6)	19 (1.2)	** < 0.0001**
Manifestations				
Rheumatoid nodules	413 (4)	339 (3.9)	74 (4.6)	0.16
Ocular	1086 (10.5)	904 (10.3)	182 (11.3)	0.37
Sjögren’s syndrome	980 (9.5)	870 (9.9)	110 (6.8)	** < 0.0001**
CNS	703 (6.8)	593 (6.8)	110 (6.8)	0.43
Vasculitis	77 (0.74)	63 (0.72)	14 (0.87)	0.26
GIT	1059 (10.2)	860 (9.8)	199 (12.3)	0.8
CVS	619 (6)	501 (5.73)	118 (7.3)	0.14
Chest	732 (7.1)	605 (6.9)	127 (7.9)	0.07
FMS	760 (7.3)	713 (8.1)	47 (2.9)	** < 0.0001**
Renal	205 (2)	137 (1.6)	68 (4.2)	** < 0.0001**
Laboratory investigations				
Hemoglobin (g/dl)	11.6 ± 1.5	11.5 ± 1.4	12.3 ± 1.7	** < 0.0001**
TLC (× 10^3^/mm^3^)	7.1 ± 2.5	7 ± 2.5	7.3 ± 2.5	** < 0.0001**
Platelets (× 10^3^/mm^3^)	297.1 ± 95.2	297.7 ± 94.8	293.1 ± 98.2	0.18
ESR (mm/1^st^ hr)	45 ± 27.7	45 ± 27.5	45.1 ± 28.7	0.85
CRP (mg/dl)	17.5 ± 22.6	16.9 ± 21.9	22.6 ± 26.4	**0.036**
ALT (IU/l)	21.7 ± 15.3	21.8 ± 15.2	21.1 ± 16	0.22
AST (IU/l)	22.6 ± 15.7	22.8 ± 15.7	21.3 ± 15.7	**0.02**
Urea (mg/dl)	19.5 ± 17.6	19.9 ± 17.6	17.5 ± 17.1	**0.009**
Creatinine (mg/dl)	0.71 ± 0.34	0.71 ± 0.32	0.76 ± 0.45	**0.001**
Cholesterol (mg/dl)	195.4 ± 62.4	193.1 ± 56.8	206 ± 87.9	**0.02**
Triglycerides (mg/dl)	123.9 ± 55.5	124.9 ± 53.7	115.8 ± 68.1	0.11
HDL (mg/dl)	55.4 ± 29.3	55.9 ± 28.8	51.5 ± 32.2	0.09
LDL (mg/dl)	104.1 ± 40.3	104.8 ± 38.7	98.6 ± 51.1	0.15
SUA (mg/dl)	4.68 ± 1.4	4.6 ± 1.4	5 ± 1.5	** < 0.0001**
RF (*n* = 7992, F:M 6877/1115)	5889 (73.7)	5022 (73)	867 (77.8)	** < 0.0001**
Anti-CCP (*n* = 5433,F:M 4617/816)	3623 (66.7)	3046 (66)	577 (70.7)	**0.007**
ANA (*n* = 2556, F:M 2271/285)	330 (12.9)	302 (13.3)	28 (9.8)	0.07
HAQ	0.95 ± 0.64	0.95 ± 0.63	0.97 ± 0.7	0.56
DAS28	4.43 ± 1.44	4.4 ± 1.4	4.3 ± 1.6	**0.015**

Statistical analysis was done using a Chi-square or Mann Whitney *U* tests. Bold values are significant at *p* < 0.05

*BMI* body mass index, *HCV* hepatitis C virus, *hx* history, *CNS* central nervous system, *GIT* gastrointestinal tract, *CVS* cardio-vascular system, *FMS* fibromyalgia syndrome, *TLC* total leucocytic count, *ESR* erythrocyte sedimentation rate, *CRP* C-reactive protein, *ALT* alanine transaminase, *AST* aspartate transaminase, *HDL* high density lipoprotein. *LDL* low density lipoprotein, *SUA* serum uric acid, *RF* rheumatoid factor, *Anti-CCP* anti-cyclic citrullinated peptide, *ANA* antinuclear antibody, *HAQ* health assessment questionnaire, *DAS28* disease activity score

**Fig. 1:**
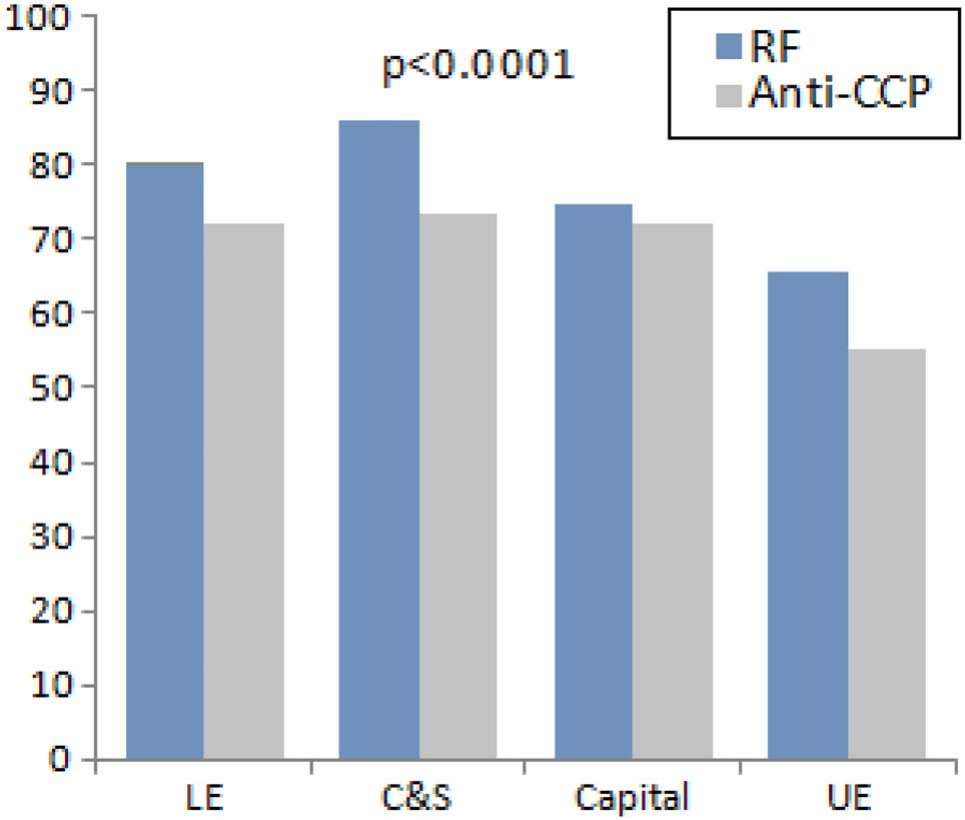
Rheumatoid factor and anti-cyclic citrullinated peptide (anti-CCP) in rheumatoid arthritis patients across different geographic regions in Egypt. *LE* lower Egypt, *C* and *S* Canal and Sinai, *UE* Upper Egypt. Statistical analysis was done using analysis of variance (ANOVA) tests

**Fig. 2:**
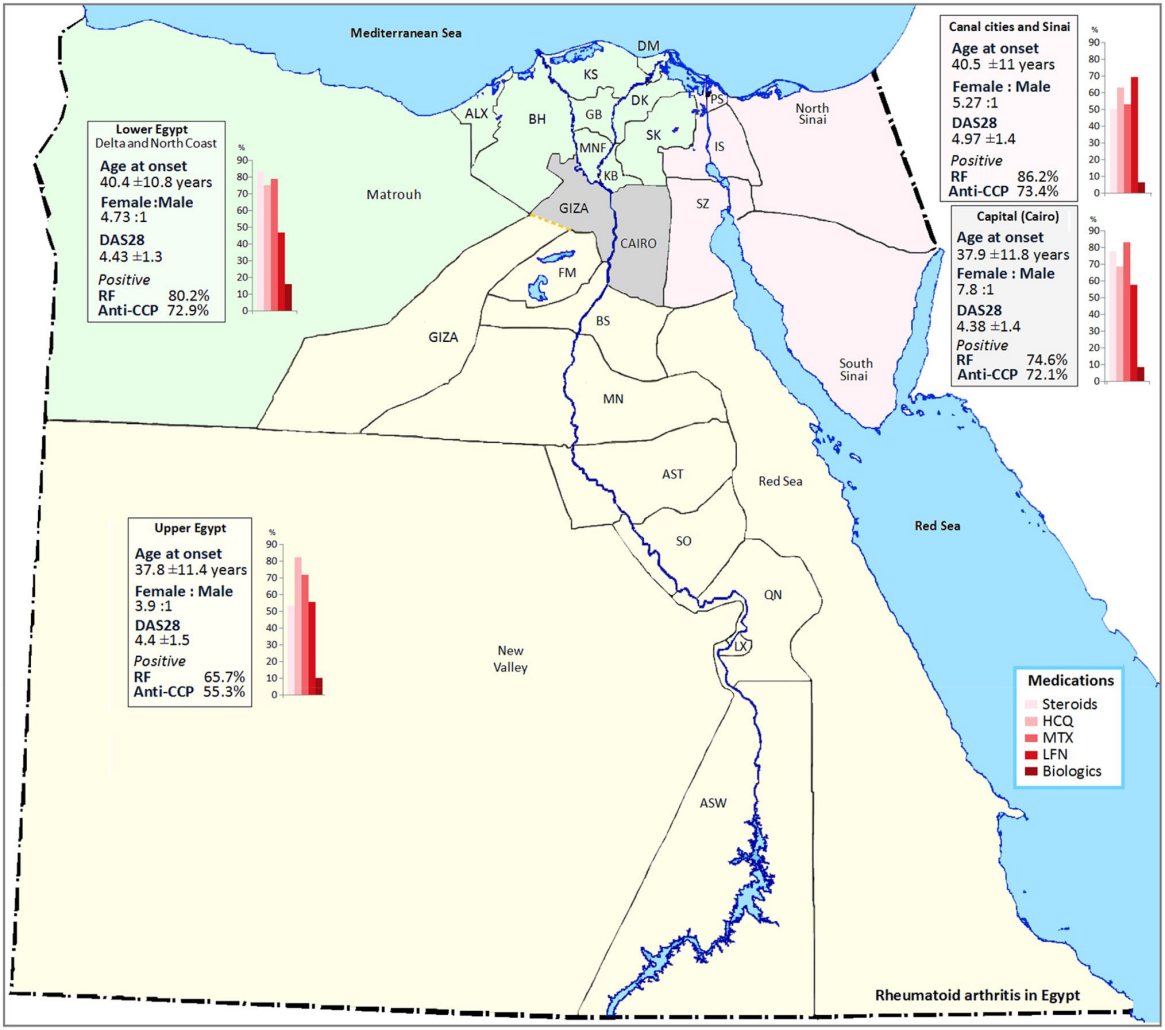
The age at onset, gender distribution, disease activity, rheumatoid factor and anti-cyclic citrullinated peptide positivity as well as the main medications received by rheumatoid arthritis patients from the four main regions across Egypt. Lower Egypt (North coast and Delta); ALX: Alexandria, BH: Beheira, KS: Kafr El Sheikh, DM: Damietta, GB: Gharbia, DK: Dakahlia, SK: Sharkia, MNF: Menoufiya, KB: Kalyoubia. Canal cities and Sinai; PS: Port-Said, IS: Ismailia, SZ: Suez. Upper Egypt; FM: Fayoum, BS: Beni-Suef, MN: Minia, AST: Assuit, SO: Sohag, QN: Qena, LX: Luxor, ASW: Aswan. HCQ: hydroxychloroquine, MTX: methotrexate, LFN: leflunomide

Certain variables according to the geo-location are presented in Table 2 and graphically presented in Figs. 1 and 2. The age at onset, gender distribution, disease activity, RF and anti-CCP positivity were significantly varied. The least age at onset, F:M, RF and anti-CCP positivity were present in Upper Egypt, while the highest DAS28 was reported in Canal cities and Sinai. The HAQ was significantly increased in Upper Egypt with the least disability in Canal cities and Sinai. Biological therapy intake was higher in Lower Egypt (46.3%), followed by the Capital (33.1%), Upper Egypt (20.3%) and the Canal cities and Sinai (0.2%) (*p* < 0.0001).

**Table 2:** Age at onset, gender, disease activity, rheumatoid factor and anti-cyclic citrullinated peptide in rheumatoid arthritis patients according to the geo-location

Geo-location		Total (*n* = 10,364)	Age at onset (years)	F:M	DAS28	Positive RF	Positive anti-CCP
Lower Egypt Delta and N Coast		1802	40.4 ± 10.8	4.73:1	4.43 ± 1.3	1204/1502 (80.2)	926/1270 (72.9)
1	Alexandria	235	35.8 ± 9.6	8.4:1	5.02 ± 1.5	88/137 (64.2)	140/189 (74.1)
2	Beheira	15	40.5 ± 9.3	4:1	4.01 ± 1.6	11/15 (73.3)	3/5 (60)
3	Kafr El-Sheikh	298	37 ± 11	4.6:1	4.8 ± 1.4	148/223 (66.4)	67/99 (67.7)
4	Damietta	59	45.2 ± 12.4	18.7:1	4.7 ± 1.2	47/59 (79.7)	13/25 (52)
5	Gharbia	558	46.4 ± 6.8	3.5:1	3.7 ± 0.82	454/513 (88.5)	469/499 (94)
6	Dakahlia	389	37 ± 11.3	4.9:1	4.6 ± 1.5	263/320 (82.2)	171/263 (65)
7	Sharkia	60	39.2 ± 10.6	7.6:1	4 ± 1.8	38/47 (80.6)	8/12 (66.7)
8	Menoufiya	53	44.8 ± 9.2	9.6:1	4.8 ± 1.1	40/53 (75.5)	30/46 (65.2)
9	Kalyoubia	135	37.5 ± 12.1	3.5:1	4.4 ± 1.03	116/135 (85.9)	25/133 (18.8)
Canal cities and Sinai		320	40.5 ± 11	5.27:1	4.97 ± 1.4	243/282 (86.2)	200/273 (73.4)
10	Port-Said	6	41.8 ± 10.7	females	4.6 ± 0.14	5/5 (100)	1/2 (50)
11	Ismailia	312	40.4 ± 10.9	5.2:1	5 ± 1.4	237/275 (86.2)	199/270 (73.7)
12	South Sinai	2	48.5 ± 30.4	1:1	4	1/2 (50)	0/1
13 Capital (Cairo)		4812	37.9 ± 11.8	7.8:1	4.38 ± 1.4	3015/4039 (74.6)	1498/2079 (72.1)
Upper Egypt		3430	37.8 ± 11.4	3.9:1	4.4 ± 1.5	1426/2169 (65.7)	999/1808 (55.3)
14	Fayoum	378	40.7 ± 11.9	5.4:1	5 ± 1.2	273/365 (74.8)	250/316 (79.1)
15	Beni-Suef	433	38 ± 11.4	3.4:1	4.9 ± 1.7	240/397 (60.5)	184/313 (58.8)
16	Minia	518	36.8 ± 10.7	4.6:1	3.7 ± 1.4	322/516 (62.4)	227/468 (48.5)
17	Assiut	823	37.7 ± 11.4	4.9:1	4.7 ± 1.5	449/706 (63.6)	260/597 (43.6)
18	Sohag	1132	37 ± 11.6	3:1	4.2 ± 1.6	35/55 (63.6)	8/16 (50)
19	Qena	42	42.7 ± 11.04	9.5:1	4.2 ± 0.97	20/30 (66.7)	7/15 (46.7)
20	Aswan	98	39.2 ± 9.4	3.9:1	4.2 ± 0.97	81/94 (86.2)	62/83 (74.7)
21	Red Sea	2	46.5 ± 7.8	Females	5.7 ± 1.1	2/2 (100)	–
22	New Valley	4	43.3 ± 11.1	Females	NA	4/4 (100)	1/2 (50)
*p*			** < 0.0001**	** < 0.0001**	** < 0.0001**	** < 0.0001**	** < 0.0001**

Statistical analysis was done using analysis of variance (ANOVA) tests. Bold values are significant at *p* < 0.05

*F:M* female to male ratio, *DAS28* disease activity score, *RF* rheumatoid factor, *ACPA*: anti-cyclic citrullinated peptide

## Discussion

This cross-sectional study presented the socio-demographic, clinical, and therapeutic profile of 10,364 RA patients recruited across Egypt. In the present work the mean age at onset of RA patients in Egypt was 38 years which was significantly lower in females. The F:M was 5.4:1. The age at onset, gender distribution and disease characteristics of RA patients in countries from different continents were compared to the current study (Table 3). Interestingly, the age at onset was lower than that in other countries and nations [10–15] while it was comparable with that from Arab countries [16] and Turkey [17]. A potential explanation could be related to the lower average age of the populations in the Middle Eastern countries [18]. However, genetic and environmental factors cannot be excluded. The higher F:M was comparable to large registries from Latin America [13, 19, 20] thus raising the subject about an increasing shift in the ratio. Once more, the BMI in the RA patients of the current study were similar to that reported from Turkey [17]. RA, the most common inflammatory rheumatic disease, is no exception, with a F:M > 4 before 50 years old and < 2 after the age of 60 [21]. Furthermore, with the increasing incidence of spondyloarthritis (SpA) worldwide, it could have been that more male patients were misdiagnosed as having RA.

**Table 3:** The age at onset, gender distribution and disease characteristics of the rheumatoid arthritis patients in countries from different continents compared to the current study

Parameter	Europe		USA	Latin		Asia			ECR
	[10]	[11]	[5, 12]	[13, 20]	[19]	[29]	[17]	[14, 15]	
no	14,438	3898	42,000	81,386	3717	4721	1038	30,501	10,364
Countries	UK	EU Canada	USA	Colombia	Brazil Argentina	South Korea	Turkey	China	Egypt
								
Centres	18	9 registries	83	39 regions	81	23	36	500 + 26 regions	26
Age at onset (ys)	≈ 43	≈45.1	≈47	≈49	≈44	43.9 ± 13	41.4 ± 13.5	≈48.6	38.4 ± 11.6
F:M	3.2:1	4.1:1	3.5:1	5.3:1	5.7:1	5.8:1	4.2:1	4.1:1	5.4:1
Smoking	21.8%	17.6%	12%		11%	8.01%	16.8%		8.2%
DAS28	6.5 ± 1	4.1 ± 1	3.5 ± 1.6	2.4 (1.8–3.3)	4.9 ± 2.7	46.9% (3.2–5.1)	3.7 ± 1.6	5.1 ± 1.7	4.4 ± 1.4
RF	–	42.5%	75%	76%	92%	86.8%		83.6%	73.7%
ACPA	–	32.7%		24%		83.9%			66.7%
MTX	56.6%	–	–	–	84.4%	81.4%	64.4%	55.9%	78%
cDMARDs				65.1%	30.3%	97.5%	90.8%		89.7%
Steroids		50.3%	38%	19%	56.7%	74%	50.4%	40.6%	71.3%
Biologics	100%	100%	100%	15.5%	69.7%	5.8%	10.5%	8.3%	11.6%

*UK* United Kingdom, *EU* Europe, *USA* United States of America, *FM* female to male, *DAS28* disease activity score, *RF* rheumatoid factor, *ACPA* anti-citrullinated peptide antibody, *MTX* methotrexate, *cDMARDs* disease modifying anti-rheumatic drugs

The misdiagnosis of SpA as RA leads to a delayed SpA diagnosis and inadequate therapeutic outcomes. Typical SpA-related clinical manifestations were present in RA patients. The advancements and accessibility of imaging modalities pave way for a more precise classification [12]. In this work, associated bronchial asthma and thyroid dysfunction, a family history of RA, Sjögren's syndrome, fibromyalgia syndrome and disease activity were significantly increased in females. It is notable that a lower frequency of females was receiving biologic therapy. On the contrary, males were significantly more smoking, had more renal manifestations, higher serum uric acid, more frequent positivity of RF and anti-CCP. Regarding the various clinical manifestations reported in this work, they were further compared to those from other countries.

Interstitial lung disease (ILD) is a well-known potentially life-threatening complication in RA [22]. The enduring appraisal of the complex relationships between smoking, COPD, and other factors in RA-associated ILD is important [23]. In this work, the reported frequency of smoking in RA patients was lower (8.2%) than that from other studies from the UK (21.8%) [10], European Union (EU) and Canada (17.6%)[11] as well as Turkey (16.8%) [17].

In this work, neurological manifestations were reported at a low frequency. The frequencies of depression and anxiety were doubled in early RA than in long-standing disease. RA patients with short disease duration and functional limitation were more likely to suffer from depression and anxiety [24].

In this study, the reported frequency of cardiovascular manifestations was low. However, there is a considerable rise in mortality and morbidity in RA due to cardiovascular disease (CVD). The augmented risk for heart disease is related to disease activity and chronic inflammation with traditional risk factors and RA-related characteristics playing a central role [25]. RA patients had higher rates of obesity than the general population and this was strongly associated with physical dysfunction [26]. The BMI in this work was higher than that reported from other nations such as the UK [10] and EU [11]. Compared to osteoarthritis (OA), RA patients were significantly more frequently diabetic and smokers but had lower prevalence of obesity and dyslipidemia [27]. The frequency of metabolic syndrome in RA patients is doubled and raises the risks of stroke and heart disease [28]. The frequency of diabetes mellitus in this work was similar to the USA [12] and Latin American [19] registries, CVD was comparable to the USA CORRONA study [12] and chest involvement was in line to the Korean registry (KORRONA)[29].

In this work, the RF was positive in 73.7% while the anti-CCP was positive in 66.7%. The frequency of RF was comparable to that from a large Colombian study on 68,247 cases [13] and to the CORONNA study from USA [12]. It was lower than Asian studies from Korea (86.8%)[29] and China (84.7%)[14]. Moreover, the frequency of anti-CCP positivity was lower than that reported in a Korean work (83.9%)[29] but higher than the registries from Colombia (24%)[13] and from the EU (32.7%)[11]. Anti-CCP and RF combined detection improves the diagnostic efficiency of RA, providing a potential strategy for early clinical screening [30]. The frequency of remission is three times higher in sero-negative patients with RA. However, the rate of remission does not depend on the serological status as almost two thirds of patients achieve remission in the first 6 months of DMARDs therapy. Anti-CCP and RF titers at the onset of the disease do not influence remission [31].

There was moderate disability in the present cases as measured by the HAQ. The functional capacity (physical and psychosocial) is a central treatment aspect to consider when the RA therapeutic strategy is personalized [32]. The average HAQ score reported in a population-based study was 0.49, and in RA was 1.2 [9]. The disease activity score in the present work was similar to that reported from the EU [11], higher than that from Turkey [17] and the USA [12] while it was lower than that from the UK [10] and China [14].

The medications received by the patients of the current study were diverse. In this study, more males were receiving HCQ and biologic therapy and with a lower disease activity. In early RA, targets can be achieved when the baseline level of diseases activity is low, with male gender and shorter disease duration [33]. In this work, MTX was received by 77.9%. Using MTX before initiating biologic therapy may contribute to a cost-effective RA care [34]. Variables related to MTX failure such as female gender, higher BMI, smoking, higher disease activity and diabetes can aid in predicting the disease process and outcome of treatment [35]. 54.8% of cases received leflunomide while 37.1% received sulfasalazine. Leflunomide is comparable to sulfasalazine in MTX-failed RA patients with similar safety profile [36]. 11.6% of the current patients were on biologics while in Korea a 6 times fold usage was reported [37].

Across the country there was a significant difference in the age at onset, gender distribution, disease activity, RF and anti-CCP positivity. A potential converse causal link between educational accomplishment and the risk of RA has been noticed [38].

National Registries are essential to direct current practice. RA registries in the Middle East and North Africa (MENA) region are rarely presented [39]. On comparing the findings to countries from other continents, variations were easily noted.

In a study from Morocco on 225 RA cases, the age of onset (44 years), F:M (7.1:1), DAS28 (5.2 ± 1), RF positivity (90.5%), anti-CCP positivity (88.8%) were higher than the current findings however, those patients were all receiving biologic therapy [40].

In a study on 300 RA patients from Palestine, treatment with biologic therapy, younger age, having work, higher income, absence of morning stiffness and absence of co-morbidities were significantly associated with better quality of life and less disability [41]. In the work from a tertiary care hospital in KSA on 288 RA patients, the majority (88%) were females with a F:M 7.3:1. In agreement to this work, hypertension was the most common co-morbidity followed by diabetes and almost all of their patients had high disease activity at presentation time [42]. Compared to patients in Western countries, South Korean patients with RA, even those with better physical function, seem to have a lower quality of life [43]. In a study conducted by the Korean College of Rheumatology (KCR) on 2422 patients with a F:M 6.8:1, 19.4% were overweight and 16.1% obese, 13.6% smoked, 11.6% had dyslipidemia, 28% were hypertensive and 4.5% were diabetic. RF and anti-CCP were positive in 82.6% and 86.9%, respectively. The mean DAS28 was 4.7 ± 1.6, 79.9% were receiving steroids, 93.2% MTX, 68.8% HCQ and 46.3% LFN while 61.7% were on biologics [37].

In a large RA registry in the UK, of 27,607 patients, 70.6% were female (F:M 2.4:1) and their mean BMI was 27.3 [44]. In a study from 11 registries from 9 European countries: France, Sweden, Czech, UK, Denmark, Italy, Germany and Portugal on 130,315 RA patients; for biologic naive patients the age at onset was 56.4 years and F:M 2.6:1 and for those who received anti-TNF the age at onset was 46.5 years and F:M was 3:1 [45].

In a large nationwide US study, the F:M was 2.4:1. Obesity was present in 15.1%, diabetes in 20.4% and dyslipidemia in 48% [46].

Although this is currently the largest data of RA patients from across Egypt, there is a desperate need for effective and applicable national management strategies and guidelines. It seems that still across the country the diagnostic tests are not strictly considered for all patients. In spite that the medications received are mostly alike among the major cities, there is a disperse intake of biologic therapy being higher along a North to South gradient.

In conclusion, the spectrum of RA phenotype in Egypt is variable across the country with an increasing shift in the F:M ratio. The age at onset was lower than in other countries.

## Data Availability

Data are available upon request.
